# Machine-actionable criteria chart the symptom space of mental disorders

**DOI:** 10.1038/s41746-026-02451-6

**Published:** 2026-02-23

**Authors:** Barbara Strasser-Kirchweger, Raoul Hugo Kutil, Georg Zimmermann, Christian Borgelt, Wolfgang Trutschnig, Florian Hutzler

**Affiliations:** 1 Department of Psychology, Centre for Cognitive Neuroscience, University of Salzburg, Salzburg, Austria; 2 Department of Artificial Intelligence & Human Interfaces, IDA Lab Salzburg, University of Salzburg, Salzburg, Austria; 3 Team Biostatistics and Big Medical Data, Paracelsus Medical University, Salzburg, Austria

**Keywords:** Computational biology and bioinformatics, Diseases, Health care, Mathematics and computing, Medical research, Psychology, Psychology

## Abstract

Diagnostic rules are codified in consensus manuals such as DSM-5, yet they remain written in narrative form and cannot be computationally interrogated. Here, a deterministic framework is presented that translates diagnostic criteria into a machine-actionable representation of the full symptom space, which can be charted, navigated, and systematically analyzed. Unlike probabilistic models that infer patterns from large textual corpora, this framework directly interrogates explicit consensus criteria, providing a transparent and reproducible means of assessing conceptual coherence. Its potential is demonstrated by charting schizophrenia-spectrum disorders, which remain conceptually distinct despite substantial symptom overlap, and by evaluating the current National Academies’ definition of Long COVID, which is largely subsumed by depressive and anxiety disorders. By making diagnostic consensus computable, the framework provides a reproducible foundation for evaluating delineation properties of existing and candidate diagnostic constructs and for developing interpretable, regulatory-compliant diagnostic support tools.

## Introduction

Accurate diagnosis depends on clear, consistent rules. Diagnostic manuals such as the *Diagnostic and Statistical Manual of Mental Disorders, Fifth Edition (DSM-5)*^1^ encode rule-based criteria that define which combinations of symptoms warrant each diagnosis. These manuals embody a normative, community-endorsed consensus that has developed over time through structured deliberation and revision, converging on a set of categories that pragmatically summarize clinical presentations, while not exhaustively capturing the full space of mental phenomena^2,3^. In the present work, we treat such manuals as operational reference standards whose explicit criteria can be formally interrogated, in principle, the proposed approach is applicable to any diagnostic manual. Yet the systematic management of these diagnostic rules remains challenging. For example, in DSM-5, each criterion gives rise to a set of valid symptom constellations, subsequently referred to as criteria-satisfying symptom combinations (CSSCs). Each CSSC is a set of symptoms that satisfies the diagnostic criteria of a disorder. In this way, CSSCs allow to decompose each diagnosis into the set of symptom configurations (though do not introduce an additional layer of abstraction on top of diagnostic labels).

Symptoms are shared across disorders, and individual disorders can be defined by an enormous number of permissible CSSCs, making the diagnostic process combinatorially explosive and beyond unaided human cognition^4,5^. Major depressive disorder (MDD) illustrates this challenge. The DSM-5 *A criterion* requires at least five of nine symptoms, one of which must be either depressed mood or loss of interest or pleasure. Even this single criterion yields more than seven million valid CSSCs. Clinicians must navigate these CSSCs not only within MDD but also across other disorders in which symptoms such as fatigue or sleep disturbance are also diagnostically relevant (e.g., anxiety disorders, bipolar disorder, and certain personality disorders^1,6^). This symptom overlap (resulting in similar CSSCs) and the enormous combinatorial breadth complicates differential diagnosis and increases the risk of misdiagnosis, ineffective treatment, and unnecessary healthcare costs^7^.

Against this background, computational methods have been proposed to support diagnostic reasoning. Large language models (LLMs) achieve strong benchmark performance; however, Singhal and colleagues^8^ report alignment with expert consensus in only about 79.5% of cases, with clinically significant errors. LLMs infer patterns from vast text corpora^8^ and approximate rather than instantiate diagnostic consensus, and their inner workings remain opaque. As Joyce and colleagues argue, psychiatry requires models that are both transparent and interpretable^9^.

Consensus criteria remain indispensable, yet although recent revisions to DSM-5 and ICD-11 have increased specificity and clinical utility^10,11^, diagnostic rules remain encoded in prose, which precludes computational access. Standardization resources such as SNOMED Clinical Terms (SNOMED CT), ICD mappings, and the Human Disease Ontology address terminology and semantic interoperability^12–14^. For example, SNOMED CT provides hierarchies and relations that support inference in decision-support systems, and is employed for higher-level decisions related to case and care management (though not diagnostic decision support^15^). Likewise, the Human Disease Ontology^16,17^, formulated in OWL, facilitates semantic interoperability and data integration. Another framework that seeks to understand and investigate mental disorders at a more mechanistic, dimensional level than the summarized definitions in DSM-5 (or other diagnostic manuals) is the Research Domain Criteria^18^ (RDoC). RDoC is primarily intended to guide research that may inform future revisions of diagnostic systems, rather than to represent the current disorder definitions used in clinical practice. None of these resources systematically represents the combinatorial, criteria-level logic of diagnostic rules as specified in existing manuals.

A machine-actionable representation of diagnostic criteria would enable the systematic identification and formal analysis of CSSCs and allow navigation of the entire diagnostic space. Here we present such a framework, which deterministically generates all CSSCs defined by narrative DSM-5 criteria. Crucially, the framework involves no machine learning or data-driven inference but relies entirely on the explicit, rule-based structure encoded in the diagnostic narrative. We demonstrate its use by confirming delineation among schizophrenia-spectrum disorders.

and by evaluating the conceptual overlap between Long COVID and established DSM-5 disorder definitions. For emerging diagnostic proposals such as Long COVID, a systematic evaluation of definitional overlap can inform the development of criteria and help determine whether a new construct is conceptually distinct. Our aim is not to revise existing DSM-5 categories, but to treat DSM-5 consensus criteria as a fixed reference against which emerging diagnostic proposals, such as Long COVID, can be systematically evaluated for delineation.

By making consensus-based diagnostic knowledge computable, our framework has dual implications: it offers a tool for refining diagnostic manuals and provides a foundation for interpretable, real-time diagnostic decision support. Explainability and transparency—qualities required by regulators and essential for clinical trust—are intrinsic to this rule-based instantiation of consensus.

## Results

### From narrative to formal representation

In order to create a machine-actionable representation of diagnostic knowledge, the descriptive language used in diagnostic manuals must be translated into formal representations. Specifically, symptoms and diagnostic criteria described in prose—for instance, in the DSM-5—must be transformed into logical structures specifying which symptoms are required or irrelevant for each disorder.

**Fig. 1:**
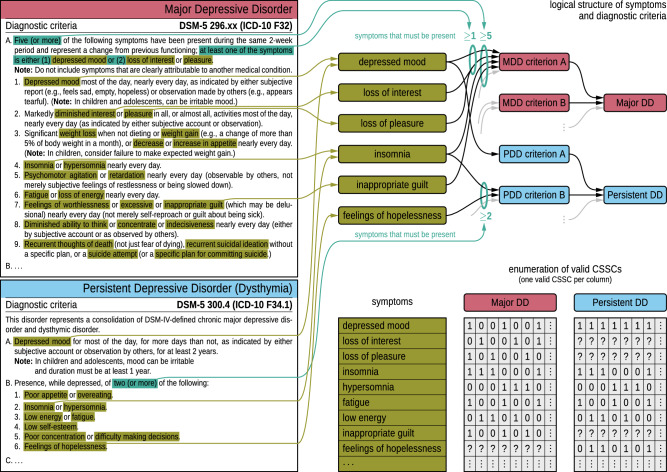
Extraction and formalization of diagnostic criteria for Major Depressive Disorder (MDD) and Persistent Depressive Disorder (PDD). **Left panels:** Narrative diagnostic criteria provided in diagnostic manuals, such as DSM-5, are analyzed to identify key elements. Exemplary criteria are shown for Major Depressive Disorder (MDD, red) and Persistent Depressive Disorder (PDD, blue). The analysis involves extracting essential components, such as specific symptom counts (e.g., “five or more”, highlighted in teal) and individual symptoms (e.g., “depressed mood” or “loss of interest”, highlighted in olive). **Upper right panel:** The afore-mentioned elements are formalized within a structured framework, where symptoms, criteria, and disorders are depicted as distinct nodes, and numerical thresholds are represented as edges connecting specific symptoms to diagnostic criteria. This framework organizes the relationships between symptoms, diagnostic criteria, and disorders. Edges illustrate the rules that define these relationships, such as how a symptom connects to a specific criterion and how that criterion links to the broader disorder. For instance, when two disorders share a symptom, the same symptom node will have rules linking it to different criterion nodes for each disorder. This structured representation enables a clear depiction of overlapping criteria and symptoms across disorders, facilitating a systematic analysis of their interrelationships. **Lower right panel:** Binary encoding of the CSSCs of the two disorders. For MDD the vector in the first column represents a CSSC: the vector indicates symptoms that are present (1), those that are absent (0), and those that are irrelevant (“?”).

To illustrate, Fig. 1 (left panels) presents excerpts of the narrative diagnostic criteria for Major Depressive Disorder and Persistent Depressive Disorder, which define conditions under which specific symptom combinations are sufficient for diagnosis. These criteria are formalized into logical statements in Fig. 1 (upper right panel). For example, the requirement in Persistent Depressive Disorder that “two (or more)” of six symptoms be present is expressed as a cardinality constraint (e.g., “≥2”) in our formal language.

Importantly, symptoms not explicitly required by a disorder’s diagnostic criteria should not be interpreted as requiring absence. Their presence or absence carries no diagnostic value unless explicitly stated. For instance, “feelings of hopelessness” is not listed among the diagnostic criteria for Major Depressive Disorder. A patient may or may not report this symptom, but it does not affect the diagnosis. In our framework, such symptoms are considered *irrelevant* for that disorder (for details on formal definitions of relevance and redundancy, see Methods).

These logical structures, which constitute the intensional definition of a disorder, enable the systematic generation of all valid CSSCs. CSSCs can be represented as binary vectors, with each dimension corresponding to a symptom: a value of 1 indicates presence, 0 indicates absence, and “?” denotes a diagnostically irrelevant symptom (see Fig. 1, lower right panel). To maintain binary integrity, irrelevant symptoms are implicitly treated as 0 during analysis (see Methods for details and Supplementary Information Section 5 for an example).

**Fig. 2:**
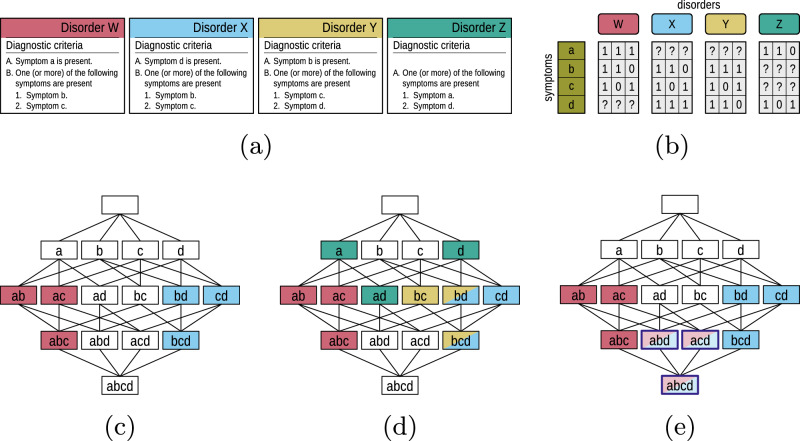
Conceptual illustration of criteria-satisfying symptom combinations (CSSCs) across four hypothetical disorders. **a** Narrative descriptions of diagnostic rules for four hypothetical disorders: W, X, Y, and Z. **b** Binary vector encodings of valid criteria-satisfying symptom combinations (CSSCs) for each disorder. Columns are grouped by disorder; rows correspond to symptoms. Values of 1 and 0 denote relevant symptoms that are present or absent, respectively. Irrelevant symptoms are indicated by “?”. **c** The full four-dimensional symptom space visualized as a Hasse diagram, showing all possible symptom combinations. CSSCs for Disorders W (red) and X (blue) are overlaid. Irrelevant symptoms are implicitly treated as 0 (see Methods for details). **d** Same symptom space, now including CSSCs for Disorder Y (yellow) and Disorder Z (teal). Weakly irredundant CSSCs (bd and bcd) shared by Disorders X (blue) and Y (yellow) violate the *no-overlap requirement* (absence of identical CSSCs), indicating that the disorders are not conceptually separable, as they share at least one identical CSSC. Symptoms a and d, representing CSSCs of Disorder Z (teal), do not overlap with CSSCs of other disorders but are *subsumed* by CSSCs of W (ab, ac, red) and X (bd, cd, blue), respectively-illustrating violations of the *no-subsumption requirement*. **e** Illustration of real-world patient presentations for Disorders W and X. Solid-colored nodes represent presentations corresponding with CSSCs, lighter nodes with dark outlines reflect presentations that include additional, non-required disorder-wise (diagnostically) irrelevant symptoms.

To further illustrate, consider a toy model with four possible symptoms: a, b, c, and d (see Fig. 2). Suppose that Disorder W requires (A) Symptom a and (B) at least one of b or c (Panel a). This yields three valid CSSCs: (1, 1, 1, ?), (1, 1, 0, ?), and (1, 0, 1, ?), where Symptom d is irrelevant and thus marked with “?” (Panel b). Disorder X, in contrast, requires (A) Symptom d and (B) at least one of b or c, resulting in (?, 1, 1, 1), (?, 1, 0, 1), and (?, 0, 1, 1), with Symptom a being irrelevant.

All possible combinations of these four symptoms define a four-dimensional symptom space, with each symptom corresponding to one dimension. Panel (c) uses a Hasse diagram to visualize this space, ranging from the empty set (top node) to the full set of symptoms (bottom node labeled “abcd”). Within this space, each disorder defines a subset corresponding to its valid CSSCs. This is illustrated for the subsets of Disorders W (red) and X (blue), overlaid on the full symptom space. Irrelevant symptoms (e.g., d for Disorder W, a for Disorder X) play no role in determining diagnostic membership and are implicitly treated as 0 (see Methods for details) when calculating, e.g., the degree of similarity of two disorders. Formally, let *U* denote the set of all possible symptom combinations in this space, and let each disorder *D* be represented by a set *S*_*D*_ ⊆ *U* of its weakly irredundant CSSCs. Thus, Disorders W and X correspond to the sets ${S}_{{\mathsf{W}}}$ and ${S}_{{\mathsf{X}}}$ of their weakly irredundant CSSCs. At this level of description, delineation between disorders can be expressed in terms of the set-theoretic relations between their respective CSSC sets.

### Delineation of disorders

In Fig. 2c, the nodes representing Disorders W and X correspond to *weakly irredundant* CSSCs— symptom combinations that exclude diagnostically irrelevant symptoms (see Methods for formal definition). These CSSCs form the basis for evaluating whether two disorders can be conceptually delineated—that is, whether their diagnostic criteria are sufficiently distinct to support reliable differential diagnosis.

To formalize this evaluation, we introduce two delineation requirements. The first is the *absence of identical CSSCs* (in the following: *no-overlap requirement*): two disorders should not share any weakly irredundant CSSCs. If such a CSSC were assigned to both disorders, it would be impossible to uniquely determine the diagnosis from that combination, thereby rendering differential diagnosis conceptually impossible. Such a case would reflect a structural flaw in the definitions themselves, as a single symptom profile would simultaneously fulfill two disorder definitions. In set-theoretic terms, the *no-overlap requirement* demands that, for two disorders such as W and X, their CSSC sets satisfy ${S}_{{\mathsf{W}}}\cap {S}_{{\mathsf{X}}}={\rm{\varnothing }}$. If a minimally sufficient configuration belonged to both ${S}_{{\mathsf{W}}}$ and ${S}_{{\mathsf{X}}}$, the mapping from symptom configurations in *U* to disorders would no longer be single-valued, and the disorders would be extensionally indistinguishable at this level of description. In other words, given the information encoded in the criteria, no further symptom assessment could distinguish which of the two disorders is present for that configuration.

In the case of Disorders W and X, see Fig. 2c, each CSSC node in the Hasse diagram is assigned to at most one disorder—either red for W or blue for X, but never both. Although the disorders share Symptoms b and c, they differ in requiring either a (for W) or d (for X), implying that no weakly irredundant CSSC is shared. Thus, disorders W and X satisfy the *no-overlap requirement*. To illustrate a failure of the *no-overlap requirement*, consider Disorder X (blue) and Y (yellow) presented in Fig. 2d. X and Y share two weakly irredundant CSSCs: bd and bcd, indicating that differential diagnosis is logically impossible based on the current definitions. These lie in the intersection ${S}_{{\mathsf{X}}}\cap {S}_{{\mathsf{Y}}}\ne {\rm{\varnothing }}$, indicating that differential diagnosis is logically impossible based on the current definitions.

The second delineation requirement is the *absence of subsumption* (in the following: *no-subsumption requirement*): there must be no pair of weakly irredundant CSSCs *c* ∈ *S*_*A*_ and *d* ∈ *S*_*B*_ such that *c* ⊂ *d* or *d* ⊂ *c* (see Methods for a formal definition). Intuitively, delineation fails whenever a weakly irredundant CSSC for one disorder is strictly contained within a weakly irredundant CSSC for another.

In the toy example, this means that the weakly irredundant CSSCs for one disorder occupy “prefix” positions in the Hasse diagram relative to those of another disorder: a configuration that suffices for the first is embedded within configuration(s) that suffice for the second. In such cases, the additional symptoms offer no meaningful distinction, and diagnostic boundaries collapse. This requirement is asymmetric and must be assessed from the perspective of each disorder individually.

As shown in Fig. 2c, no weakly irredundant CSSC for Disorder W is a strict subset of any for Disorder X, and vice versa. This is visually represented in the Hasse diagram: no node of one disorder lies directly beneath a node of the other, connected via an edge. Accordingly, Disorders W and X also fulfill the *no-subsumption requirement*. Panel (d) illustrates a violation of the *no-subsumption requirement* for Disorder Z in green with respect to W and X. In fact, we can find weakly irredundant CSSCs of Z, namely a and d, which (while not being CSSCs of W and X) are strict subsets of CSSCs of W and X.

Due to the asymmetry of the *no-subsumption requirement*, this subset relationship compromises delineation even if the reverse does not hold. When such subset relationships were to arise in both directions—that is, when the minimally sufficient configurations for each disorder are fully embedded as strict subsets of configurations of the other—the violation of non-subsumption would collapse into a failure of the *no-overlap requirement*, indicating complete mutual indistinguishability. Conversely, if neither overlap nor one-sided subsumption is present, the corresponding disorders occupy distinct, non-nested regions of the CSSC space, which is the notion of conceptual separability that our formalization is designed to capture.

When conceptual delineation is not achievable, the unavoidable resulting diagnostic ambiguity calls into question the validity and coherence of the disorder definitions. Such cases suggest the need for conceptual refinement or the identification of additional, as-yet-undefined symptoms enabling reliable diagnostic separation.

It is also important to distinguish failures in conceptual delineation from the variability observed in real-world patients. Patients may exhibit additional symptoms beyond those required for diagnosis, or may meet criteria for multiple disorders when different sets of required symptoms are present. This distinction is illustrated in Fig. 2e: a patient presenting with all four symptoms (a, b, c, and d) would meet the criteria for both Disorder W and Disorder X (they would, in fact, meet the criteria for all four disorders). Since each disorder treats one of those symptoms as irrelevant, both diagnoses would be valid and reflect comorbidity — not a failure of conceptual delineation.

To summarize, the approach outlined so far transforms narrative diagnostic criteria into a formal, machine-actionable framework enabling the systematic generation of all valid CSSCs for each disorder represented in the system. These combinations constitute a disorder’s derived combinatorial structure, providing a basis for rigorously evaluating the internal logic and conceptual coherence of diagnostic definitions, and for assessing whether two disorders are formally delineable (based on the absence of shared or subsumed CSSCs). Having access to the complete set of CSSCs for two disorders also enables quantitative comparisons, allowing us to move beyond binary delineability and measure the degree of similarity between disorders based on their weakly irredundant CSSCs, encoded as binary vectors.

### Quantifying conceptual relatedness

To quantify the degree of similarity between two disorders, we compute the Maximum Pairwise Cosine Similarity (MPCS) between their respective weakly irredundant CSSCs. Importantly, all operations in this framework are deterministic and rule-based: they act directly on the explicit symptom criteria without any parameter learning from data. As a result, every intermediate object (e.g., sets of CSSCs, overlap/subsumption relations, and pairwise similarity values) can be inspected and interpreted directly, rather than being opaque in the sense of typical machine-learning models. Cosine similarity yields a similarity score between 0 and 1, where 1 indicates identity and 0 indicates no shared symptom presence. While a full mathematical description of the procedure is provided in Methods, the key steps are as follows: Given two disorders, X and Y, characterized by their sets of weakly irredundant CSSCs, we: (i) compute, for each CSSC in X, its maximum cosine similarity to any CSSCs in Y; (ii) aggregate these similarity values by either taking the mean or the maximum over the CSSCs of X; (iii) repeat the same procedure in reverse—from Y to X; (iv) take the maximum of the two resulting values, i.e., of the two maxima or the two means, yielding either

M*PCS*_m*ax*_ or M*PCS*_m*ean*_. Note that in order to compute cosine similarity, each CSSC must be embedded in a shared symptom space. This requires augmenting the vectors with the irrelevant symptoms from the other disorder (i.e., symptoms that are relevant for one disorder but irrelevant for the other are encoded as 0 for that disorder), ensuring a consistent dimensionality across comparisons (see Supplementary Information, Section 4 for an example).

A value of M*PCS*_m*ax*_ = 1 indicates the presence of at least one identical CSSC between X and Y, violating the *no-overlap requirement*. Conversely, a value of 0 implies that the sets of relevant symptoms are completely disjoint — only in this case can both M*PCS*_m*ax*_ and M*PCS*_m*ean*_ be zero. In all other cases, the presence of shared symptoms will necessarily produce nonzero similarity values, even when disorders are otherwise delineable (see Table 1).

**Table 1:** Obtained pairwise MPCS_mean_ values for the disorders mentioned in Quan-tifying conceptual relatedness

Zero similarity was observed between Speech Sound Disorder and each of the schizophrenia spectrum disorders, confirming their conceptual separation: A value of zero indicates that the sets of symptoms relevant for diagnosing one disorder are entirely disjoint from those of the other. By contrast, a gradient of similarity was observed within the schizophrenia spectrum, with the highest MPCS_mean_ value found between Schizophrenia and Schizophreniform Disorder.

Turning to our toy example, M*PCS*_m*ean*_ values are (W, X) = 0.556, (W, Y) = 0.661, (X, Y) = 0.939, (W, Z), (X, Z) = 0.664, and (Y, Z) = 0.426 (see also Table 3). Disorders X and Y exhibit the highest *average* similarity, consistent with their shared CSSCs in Fig. 2.

To validate the proposed similarity measure, we applied our approach to a selected set of disorders within the schizophrenia spectrum—specifically, Delusional Disorder, Schizophreniform Disorder, Schizophrenia, and Schizoaffective Disorder — as defined by the DSM-5. To provide contrast, we included Speech Sound Disorder as a conceptually unrelated control. For each disorder, we enumerated the complete set of valid CSSCs. These were encoded as binary vectors, with dimensionality determined by the total number of unique symptoms involved.

Table 1 presents the M*PCS*_m*ean*_ values for the selected disorders and confirms the expected conceptual separation of Speech Sound Disorder, which exhibited zero similarity with all schizophrenia spectrum disorders. This result serves as a plausibility check for our framework: a similarity score of zero implies that the sets of symptoms relevant for diagnosing the two disorders are entirely disjoint.

By contrast, the highest similarity was observed between Schizophrenia and Schizophreniform Disorder (M*PCS*_m*ean*_ = 0.744), consistent with their close clinical and diagnostic relationship. Both disorders share nearly identical core symptom requirements —such as delusions, hallucinations, and disorganized speech — with the primary distinction being the duration of symptoms required for diagnosis^1^. As a result, many CSSCs for one disorder closely resemble those of the other. This structural similarity reflects their clinical proximity as discussed below.

Building on these findings, the next section assesses whether such conceptually related disorders — notably Schizophrenia and Schizophreniform Disorder —not only exhibit high structural similarity but also fulfill the formal requirements for conceptual delineation.

### Delineation of established disorders

Building on our previous finding that Schizophrenia and Schizophreniform Disorder exhibit high structural similarity in their CSSCs, we now evaluate whether these two disorders fulfill the delineation requirements defined in Delineation of disorders. While their shared core symptom requirements account for the observed similarity, a failure to meet the delineation criteria would indicate a conceptual redundancy, precluding clear differential diagnosis.

As before, the analysis is based on the fully enumerated sets of weakly irredundant CSSCs for both disorders. To test the *no-overlap requirement* — the absence of identical CSSCs — we again applied the MPCS. Specifically, we computed M*PCS*_m*ax*_ values to identify whether any pair of CSSCs — one from each disorder — was fully identical (see Methods for details).

For the hypothetical disorders introduced in Fig. 2, M*PCS*_m*ax*_ values were: (W, X) = 0.667, (W, Y) = 0.816, and (W, Z), (X, Z), (Y, Z) = 0.707 (see Table 3). The pair (X, Y) yielded a value of 1.000, indicating a failure of the *no-overlap requirement* due to at least one identical CSSC — consistent with the overlap depicted in Fig. 2.

**Fig. 3:**
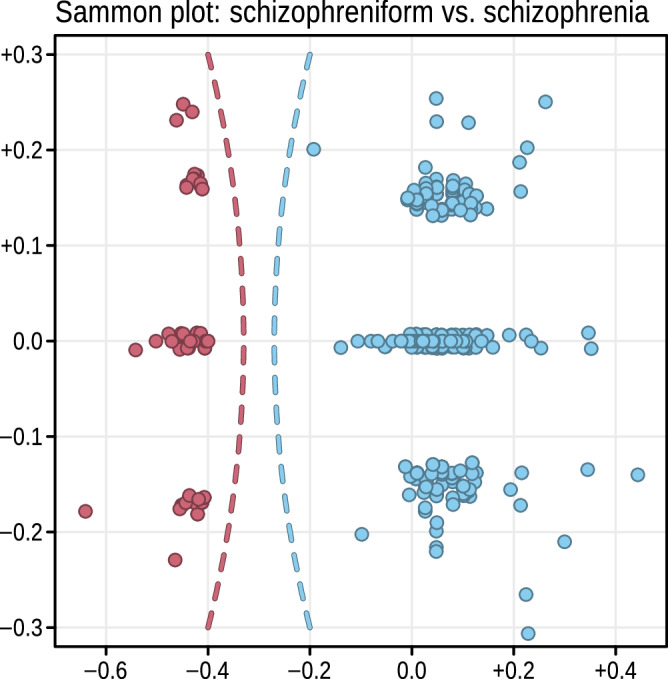
Sammon projection of CSSCs for Schizophrenia and Schizophreniform Disorder. Sammon projection of the high-dimensional CSSCs for Schizophrenia (blue) and Schizophreniform Disorder (red), mapped into a two-dimensional space while preserving pairwise distances. The clear spatial separation between the two sets of binary vectors supports their conceptual delineation and illustrates that no identical CSSCs are shared between the disorders.

In contrast, for Schizophrenia and Schizophreniform Disorder, M*PCS*_m*ax*_ = 0.800, confirming that no identical CSSCs exist between the two disorders. The *no-overlap requirement* is therefore satisfied. Figure 3 visualizes the relationship between CSSCs of the two disorders using a Sammon projection, which embeds high-dimensional vectors — such as binary vectors in our case—into two dimensions while preserving pairwise distances (see Methods). The resulting projection illustrates a clear separation between the combination sets, demonstrating fulfillment of the non-overlap criterion.

To test the *no-subsumption requirement* — the absence of subsumption — we evaluated whether any weakly irredundant CSSC from one disorder was a strict subset of a CSSC from the other. Because subsumption is not symmetric, both directions were assessed separately. The analysis revealed that no CSSC from Schizophrenia was a subset of any from Schizophreniform Disorder, and vice versa. Thus, the *no-subsumption requirement* is also fulfilled.

Finally, we examined which features accounted for delineation despite substantial symptom overlap. The key differentiating factor was symptom duration: Schizophrenia requires symptoms to persist for at least six months, whereas Schizophreniform Disorder is defined by a duration between one and six months. Schizophreniform Disorder is often diagnosed provisionally and may transition into Schizophrenia if symptoms persist beyond six months, a continuity that is well-documented both conceptually and epidemiologically^1,19–21^.

While such an analysis remains tractable for pairwise comparisons, the computational complexity increases substantially when evaluating delineation across multiple disorders simultaneously — a challenge we revisit in the Discussion.

Our findings confirm that Schizophrenia and Schizophreniform Disorder satisfy both delineation requirements: they share neither identical nor subsumed CSSCs. This ensures that differential diagnosis is possible based solely on their formalized criteria. More broadly, this example illustrates how our framework enables the systematic evaluation of diagnostic coherence by leveraging the combinatorial structure of disorder definitions.

### Newly emerging clinical conditions

Having demonstrated how our approach can quantify similarity and assess delineation among established disorders, we now apply it to a newly emerging clinical condition: Long COVID. Characterized by persistent and often debilitating symptoms following acute SARS-CoV-2 infection, Long COVID currently lacks community-established, iteratively refined diagnostic criteria.

A recent consensus report by the U.S. National Academies of Sciences, Engineering, and Medicine (NASEM) defines Long COVID as a chronic, systemic disease state persisting for at least three months and involving one or more organ systems^22^. Common symptoms include fatigue, cognitive dysfunction, and mood disturbances^23^—features also observed in established disorders such as MDD, raising the potential for diagnostic ambiguity. The NASEM report emphasizes that “no published, standardized guidelines for developing disease definitions” currently exist for Long COVID^22^. Instead, the proposed definition synthesizes multiple sources and emphasizes attribution, time course, clinical features, and exclusions. While appropriate for an evolving condition, this approach underscores the need for formal tools to evaluate whether emerging disorder definitions are conceptually distinct.

Long COVID presents a compelling test case for our framework. The definition proposed by Ely et al.^23^ organizes Long COVID into three conceptual levels.

Level 1 specifies symptoms or symptom clusters commonly reported in affected individuals. The original wording describes these as: *"Single or multiple symptoms, such as shortness of breath, cough, persistent fatigue, postexertional malaise, difficulty concentrating, memory changes, recurring headache, lightheadedness, fast heart rate, sleep disturbance, problems with taste or smell, bloating, constipation, and diarrhea.”* ^23^, p. 1747. To align these terms with DSM-5 terminology, minor phrasing adjustments were required. For example, “difficulty concentrating” was reformulated as “diminished ability to concentrate” (with “poor concentration” used as a synonym). The complete mapping from the original terms to their DSM-5 equivalents is provided in the Supplementary Information (Section 4). Level 2 addresses co-occurring diagnosable conditions—including cardiovascular disease, arrhythmias, and mood disorders—which are treated either as differential diagnoses or comorbidities. This level provides a natural comparison point: we assess whether the symptom combinations implied by Long COVID are formally delineable from those of established disorders in this set. Level 3 captures broader contextual features of the condition, including diagnostic setting, applicability across age groups, and its relapsing-remitting or progressive course. While clinically relevant, these features are not formally encoded in our combinatorial framework and thus fall outside the scope of the present analysis.

The central question is whether the symptom combinations implied by Long COVID’s Level 1 definition give rise to a set of CSSCs that are formally delineable from those of established disorders, particularly those listed as differential diagnoses in Level 2.

To evaluate the *no-overlap requirement* (i.e., the absence of identical criteria-satisfying combinations), we compared the set of CSSCs derived from Level 1 Long COVID definition to those of nine established DSM-5 disorders spanning neurodevelopmental, psychotic, depressive, and anxiety-related categories (see Symptom harmonization for details). Following the procedure introduced earlier, we computed the Maximum Pairwise Cosine Similarity (MPCS) for each disorder pair. Specifically, we used M*PCS*_m*ax*_ to identify whether there is at least one identical combination. Table 2, column M*PCS*_m*ax*_, summarizes the results.

**Table 2:** Comparison of criteria-satisfying symptom combinations (CSSCs) derived from the Level 1 definition of Long COVID with those of selected DSM-5 disorders to assess delineation requirements

The *no-overlap requirement* — absence of identical combinations, evaluated via the column M*PCS*_m*ax*_ — reports the maximum pairwise cosine similarity between any Long COVID CSSC and CSSCs of each comparator disorder. The no-overlap requirement is assessed via the M*PCS*_m*ax*_ column, where values below 1 indicate that no identical combinations were found. The *no-subsumption requirement*— absence of subsumption — is addressed in the subsequent columns. Column *N*_sub_ (LC) reports the number of weakly irredundant CSSCs of Long COVID that are strict subsets of at least one weakly irredundant CSSC of an established disorder and column *N*_sub_ (D) reports the number of weakly irredundant CSSC of an established disorder that are criteria-satisfying for Long COVID (i.e. are supersets of at least one weakly irredundant CSSCs of Long COVID). For reference, *N*_total_ provides the total number of weakly irredundant CSSCs for each established disorder.

Long COVID showed no similarity to schizophrenia spectrum disorders, Delusional Disorder, or Speech Sound Disorder, all yielding M*PCS*_m*ax*_ values of 0. Substantial similarity values were observed with depressive and anxiety-related disorders, including Persistent Depressive Disorder (0.816), Panic Disorder (0.816), Major Depressive Disorder (0.756), and Generalized Anxiety Disorder (0.707). Despite substantial similarity with mood and anxiety disorders, no disorder in our comparison set shared any identical CSSC with Long COVID — that is, no M*PCS*_m*ax*_ reached 1. Thus, the *no-overlap requirement* is satisfied.

To evaluate the *no-subsumption requirement*, we assessed whether any weakly irredundant CSSC of an established disorder is criteria satisfying for Long COVID, or vice versa. No subsumption was observed in either direction for Speech Sound Disorder, Delusional Disorder, or any of the schizophrenia spectrum disorders, confirming their conceptual separation from Long COVID.

In contrast, several mood and anxiety disorders violated this requirement. A small number of Long COVID combinations were strict subsets of valid CSSCs from Major Depressive Disorder (*N*_sub_ = 15), Persistent Depressive Disorder (15), Panic Disorder (15), and Generalized Anxiety Disorder (7). Conversely, over 1.29 billion of the 1.38 billion valid CSSCs for Major Depressive Disorder (94%) satisfied the Level 1 criteria for Long COVID. Similar proportions were observed for the other mood and anxiety disorders, indicating substantial conceptual overlap (see Table 2).

These findings carry significant implications for both clinical practice and research. The fact that over 90% of valid symptom combinations for major depressive and anxiety disorders satisfy the current Level-1 definition of Long COVID suggests a diagnostic framework that is too permissive to ensure conceptual distinctiveness. Without additional constraints — such as symptom chronology, exclusion logic, or biomarkers — this broad definition may overestimate prevalence and blur distinctions between clinical entities, reducing diagnostic precision in treatment and surveillance. In this light, formal delineation testing offers more than conceptual clarity: it becomes an essential safeguard against diagnostic inflation and category collapse.

We may be witnessing a case of *diagnostic drift*: although adding a history of COVID-19 infection could, in principle, restore delineation, this constraint has become practically meaningless. In a post-pandemic world, prior infection applies to the vast majority of the population and thus functions as a *trivial predicate* in the logical sense—true for (nearly) all. While such an addition might formally enforce delineation, it would do so at the expense of clinical utility.

## Discussion

This work demonstrates a proof of principle for systematically transforming narrative diagnostic knowledge — as codified in consensus manuals such as the DSM-5 — into a formalized, machine-readable, and machine-actionable representation, which can then be further analyzed. By translating diagnostic criteria into logical structures and binary vectors we enabled direct access to the full extension of disorder definitions and established the means to evaluate their conceptual delineation.

We introduced two formal delineation requirements — the absence of identical CSSCs (*no-overlap requirement*) and the absence of subsumption (*no-subsumption requirement*) — as necessary conditions for distinguishing between disorder definitions in a logically coherent diagnostic framework. Applying these criteria, we first validated the framework using established disorders, showing that Schizophrenia and Schizophreniform Disorder, despite substantial overlap in symptom criteria, fulfill both delineation requirements and can be distinguished on the basis of duration. We then applied the same framework to Long COVID as an emerging clinical condition. While no identical combinations were identified, several CSSCs derived from its candidate definition were strictly subsumed by CSSCs of existing depressive and anxiety disorders.

This asymmetry indicates that, under the NASEM Level-1 candidate definition, Long COVID does not achieve conceptual independence and highlights the need for delineation testing in the development of new diagnostic categories. More broadly, our findings show how formal representations of diagnostic criteria can expose conceptual redundancies, could inform expert-led refinement of candidate disorder definitions, supporting more rigorous, evidence-based expansion of diagnostic systems. Thus, existing consensus definitions serve as a reference standard against which emerging proposals are formally stress-tested.

The approach described here shows that a formal, machine-actionable representation of diagnostic knowledge can help clarify core conceptual challenges in diagnostic classification. While narrative diagnostic criteria are interpretable by humans, they often obscure logical relationships between disorders, making it difficult to assess whether definitions are meaningfully distinct.

By encoding diagnostic rules into logical structures and generating the full space of criteria-satisfying symptom combinations (CSSCs), this framework reveals overlaps that may remain hidden in narrative form. That emerging definitions—such as that of Long COVID—an yield combinations fully subsumed under established disorders highlights the value of rigorous, formal tools to evaluate diagnostic coherence.

These findings also expose the scale of complexity clinicians must navigate. For example, Major Depressive Disorder yields billions of valid CSSCs. Manually comparing such enormous numbers is infeasible, particularly as the number of disorders and comparisons grows. Without formalized, machine-supported representations, ensuring conceptual delineation across a growing diagnostic landscape becomes practically impossible. As the number of diagnostic categories and criteria grows, systematically assessing conceptual delineation across the diagnostic landscape becomes increasingly difficult to perform exhaustively without formal, computational support.

Crucially, the proposed framework could also be utilized as a tool by expert committees for future, iteratively refinements of diagnostic manuals. As new conditions arise and criteria evolve, formal delineation testing at the level of symptom combinations offers a scalable mechanism for validating whether a proposed definition is truly distinct — supporting the adherence to clear boundaries and a coherent classification system.

While the framework presented here provides a foundation for machine-actionable diagnostic reasoning, its implementation raises several technical and methodological challenges.

A core challenge is the translation of narrative diagnostic criteria into formal logic. Diagnostic manuals are written for clinical interpretability, not computational precision. As such, they often contain semantically ambiguous expressions—such as “weight change”, which implicitly covers both weight gain and weight loss—that are easily understood by clinicians but must be disambiguated explicitly for algorithmic processing. Effective translation therefore, requires expert-guided modeling of symptom boundaries to ensure mutual exclusivity and logical consistency.

A second challenge lies in the computational scale of the problem. For disorders with multiple symptoms, the number of valid symptom combinations grows exponentially. As demonstrated in this study, even a single diagnostic criterion can yield millions of valid CSSCs. Enumerating and storing these combinations—especially across multiple disorders—places significant demands on memory, computation, and runtime, requiring the use of high-performance computing infrastructure and tailored optimization techniques.

Beyond practical concerns, these challenges are rooted in fundamental computational theory. Determining whether two disorders share any valid symptom combination can be reduced to a variant of the propositional satisfiability problem -” a canonical NP-complete problem in computer science^24,25^.

Taken together, these challenges emphasize that machine-actionable models of diagnostic knowledge are not only technically feasible, but also scientifically non-trivial. Their successful implementation depends on careful logical modeling, rigorous complexity-aware design, and close collaboration between clinical experts and computational scientists.

Probabilistic systems, such as large language models (LLMs), are trained on vast corpora of biomedical literature and web content and reproduce statistically frequent patterns. While this makes them highly effective for information retrieval, it also creates the risk of conflating frequency with authority—externalizing the consensus process and mistaking regularities in language for explicit, community-governed standards. Despite benchmark promise, LLMs exhibit clinically significant limitations^8^, confirming that they approximate but do not instantiate expert consensus — leaving them not adequate for domains such as diagnosis, where rule-based clarity and reproducibility are essential.

By contrast, a deterministic framework encodes DSM rules directly, ensuring that results are produced only when formally justified—and abstained from otherwise—thus preventing unsupported outputs. As Joyce and colleagues^9^ emphasize, psychiatry requires systems that are transparent and interpretable rather than opaque black boxes. Crucially, diagnostic consensus is a normative construct grounded in structured expert deliberation, procedural transparency, and collective endorsement^2,3^, not a statistical artifact. The framework presented in this manuscript instantiates this consensus explicitly, enabling transparency, reproducibility, and regulatory compliance.

In this context, deterministic systems provide a necessary complement to probabilistic models. Rather than replacing consensus, this approach makes it computable—providing auditable and rule-based support grounded in formal clinical logic and directly addressing calls for transparency in AI/ML-enabled medical devices, which emphasize explainability as a prerequisite for regulatory approval and safe deployment^26^. Taken together, these advantages illustrate why deterministic approaches are not only conceptually rigorous but also practically relevant for clinical and translational applications.

The systematic representation and combinatorial analysis of diagnostic knowledge presented here create new possibilities, both for clinical practice and the development of a new field of diagnostic knowledge engineering. This framework enables a combinatorial, criteria-level analysis of symptom configurations that is not feasible to perform exhaustively by unaided human reasoning, and thereby complements clinicians’ expertise in evaluating the delineation of diagnostic constructs. Disorders like Major Depressive Disorder can yield billions of valid symptom combinations, far beyond what clinicians can reliably track, especially under time pressure. In such contexts, heuristic reasoning—while efficient—may introduce biases that compromise diagnostic accuracy^27,28^. One potential future

application is the development of evidence-based recommender systems for clinical use. They could systematically identify symptoms that are most discriminative for candidate diagnoses, guiding clinicians toward targeted, efficient, and transparent differential diagnosis. They could enable comparison of patient presentations against the full set of CSSCs across all disorders — an analytic capability that has, until now, been impractical without computational assistance. Concretely, given a patient’s current symptom pattern, such a system could identify all diagnoses that remain potential candidates (that is, diagnoses whose criteria are already satisfied by the current symptom pattern or could be satisfied if certain not-yet-assessed symptoms turn out to be present) and exclude diagnoses that are no longer compatible. It could then recommend which symptom to assess next, whose presence or absence would most efficiently narrow down the remaining diagnostic candidates.

Rather than replacing clinical expertise, this deterministic framework supports it by providing transparent, rule-based, and reproducible decision-making grounded in consensus-based descriptions of disorders that reflect the current state of professional knowledge (but do not constitute a definitive ground truth about the underlying disease entities)^29^. Although, in a potential clinical decision-support setting, these computations would be presented to users only as interpretable summaries (e.g., which diagnoses remain compatible with the current symptom pattern and which additional symptoms are most discriminative, rather than as raw vectors or similarity codes), the present framework remains anchored in explicit symptom combinations and simple logical relations, and does not depend on latent, learned representations. Such a framework could support clinicians in navigating the full diagnostic space, complementing other explainable AI systems that integrate domain knowledge into real-time diagnostic decision support^30^.

Structured systems could also serve educational purposes, exposing users to nuanced differentiations between closely related disorders and thereby improving diagnostic training. Moreover, by reducing reliance on heuristic shortcuts under time pressure, these tools may help mitigate cognitive biases and improve consistency in clinical assessments.

Beyond individual diagnosis, this formalized framework offers a foundation for expert committees to refine and update diagnostic manuals, should they elect to use such tools.

It would allow highlighting of definitional inconsistencies, and exhaustive comparisons between disorders could help expert committees evaluate the coherence of proposed criteria, identify unintended conceptual shortcomings, and clarify overlapping constructs. These capabilities also support the development of differentiated criteria early in the process of defining new disorders and promote alignment across related disorder domains.

Although this study focused on disorder definitions as encoded in narrative manuals, the framework could be extended to include diagnostic tools — such as structured interviews or symptom checklists — provided these tools are formally defined. This would enable not only the representation of disorder logic but also the efficient operationalization of next-step diagnostic procedures, guiding clinicians toward tools that best capture relevant symptoms based on a patient’s presentation.

Finally, as emerging conditions such as Long COVID illustrate, the ability to test whether a candidate disorder definition yields conceptually delineable symptom combinations is critical for ensuring that diagnostic categories remain clinically useful and scientifically valid. By codifying definitions formally and enabling precise, consensus-based reasoning, such systems reduce the risk of hallucination or normative drift — a gradual misalignment from formally accepted diagnostic standards. In this way, structured, machine-readable representations of diagnostic knowledge offer not only a path toward more reliable diagnostic decision-making but also a platform for the continuous refinement of medical classification systems.

## Methods

### Definitions of core notions

Let *S* = {*a*, *b*, *c*, *d*, … } be a fixed set of symptoms. A CSSC for a disorder is any subset *C* ⊆ *S* that fulfills the formal diagnostic criteria of that disorder. The criteria for each disorder, denoted X, Y, etc., can be defined by a logical rule over *S* that determines which subsets *C* qualify as CSSCs. The set of all CSSCs for a disorder X is referred to as its *extension*.

A CSSC *C* is called *irredundant* if no proper subset of *C* also qualifies as a CSSC. Otherwise, *C* is *redundant*. A symptom *s* ∈ *S* is considered *relevant* for disorder X if it appears in at least one irredundant CSSC of X. Otherwise, it is *irrelevant*. A CSSC is called *weakly irredundant* if it contains only relevant symptoms. It is called *strongly redundant* if it includes any irrelevant symptom. In our binary vector encodings, irrelevant symptoms are denoted by “?”, and are implicitly treated as absent during evaluation.

To assess the delineation of disorders, we define two requirements. The *no-overlap requirement* is violated if there exists at least one CSSC that is weakly irredundant for both X and Y. The *no-subsumption requirement* is violated if there exists at least one CSSC that is weakly irredundant for X and also satisfies the criteria of Y, even if not weakly irredundant for Y. In this case, disorder X is said to *subsume* disorder Y. Subsumption is asymmetric and strictly weaker than overlap.

Illustrative examples for each of these core notions, with references to figures and tables in the main text, are provided in the Supplementary Information (Section 1).

### Cosine similarity

To enable quantitative comparison of disorders, each criteria-satisfying symptom combination (CSSC) is represented as a binary vector over the shared symptom space, with 1 indicating presence, 0 absence of relevant symptoms, and “?” indicating irrelevant symptoms (implicitly treated as absent during evaluation and encoded as 0). Each disorder is thus described by a binary matrix *A* ∈ {0, 1}^*n*×*m*^, where columns correspond to CSSCs and rows to symptoms.

Similarity between two disorders is assessed via *pairwise cosine similarity*. For binary (or general) vectors *A* = (*A*_1_, …, *A*_*n*_) and *B* = (*B*_1_, …, *B*_*n*_), cosine similarity is the cosine of the angle between the two vectors and defined as

1 $${{\rm{S}}}_{{\rm{C}}}(A,B)=\frac{\langle A,B\rangle }{\parallel A{\parallel }_{2}\cdot \parallel B{\parallel }_{2}}=\frac{{\sum }_{i=1}^{n}\,{A}_{i}{B}_{i}}{\sqrt{{\sum }_{i=1}^{n}{A}_{i}^{2}}\sqrt{{\sum }_{i=1}^{n}{B}_{i}^{2}}},$$

where 〈 ⋅ , ⋅ 〉 denotes the inner product and ∥ ⋅ ∥_2_ the Euclidean norm^31^. For binary vectors *A*, *B* equation (1) reduces to

2 $${S}_{C}(A,B)=\frac{\#\{1\le i\le n| {A}_{i}=1={B}_{i}\}}{\sqrt{\#\{1\le i\le n| {A}_{i}=1\}\cdot \#\{1\le i\le n| {B}_{i}=1\}}},$$

Here, *#* denotes set cardinality. While cosine similarity generally ranges from [− 1, 1], it is restricted to [0, 1] for binary vectors. To ensure comparability, all CSSCs are embedded in a shared symptom space of equal dimensionality.

Given two binary matrices **A** and **B** representing the full sets of CSSCs for disorders X and Y, respectively, we quantify their similarity using either the maximum or the mean of pairwise cosine similarities. For simplicity, let *A*_1_, …, *A*_*m*_ denote the columns of **A** and *B*_1_, …, *B*_*l*_ the columns of **B**.

In both approaches, we first compute the Maximum Pairwise Cosine Similarity (MPCS): for each fixed column *A*_*i*_ in **A**, we calculate its cosine similarity with all columns *B*_*j*_ in **B** and retain the maximum value. Mathematically, we compute:

3 $${S}_{MC}({A}_{i},{\bf{B}})=\mathop{\max }\limits_{j\in \{1,\ldots ,l\}}{S}_{C}({A}_{i},{B}_{j})$$

for every *i* ∈ {1, …, *m*}. These highest similarities *S*_MC_(*A*_1_, **B**), …, *S*_MC_(*A*_*m*_, **B**) are then aggregated, either by taking the maximum or the mean, depending on the purpose of the computation. Specifically, we define:

4 $${\phi }_{{\rm{m}}ax}({\bf{A}},{\bf{B}})=\mathop{\max }\limits_{i\in \{1,\ldots ,m\}}{S}_{MC}({A}_{i},{\bf{B}})$$

or

5 $${\phi }_{{\rm{mean}}}({\bf{A}},{\bf{B}})=\frac{1}{m}\mathop{\sum }\limits_{i=1}^{m}{S}_{MC}({A}_{i},{\bf{B}})$$

We refer to *ϕ*_m*ax*_ and *ϕ*_m*ean*_ as maximum and mean similarity of **A** to **B**, respectively. Reversing the roles and repeating the process yields *ϕ*_m*ax*_(**B**, **A**) as well as *ϕ*_mean_(**B**, **A**). The final Maximum Pairwise Cosine Similarity is then either the quantity

6 $${{\mathrm{MPCS}}}_{{\mathrm{max}}}({\bf{A}},{\bf{B}})=\max ({\phi }_{{\rm{m}}ax}({\bf{A}},{\bf{B}}),{\phi }_{{\rm{m}}ax}({\bf{B}},{\bf{A}}))$$

or the number

7 $${{\mathrm{MPCS}}}_{{\mathrm{mean}}}({\bf{A}},{\bf{B}})=\max ({\phi }_{\mathrm{mean}}({\bf{A}},{\bf{B}}),{\phi }_{\mathrm{mean}}({\bf{B}},{\bf{A}})).$$

Obviously we always have

8 $$0\le {{\mathrm{MPCS}}}_{{\mathrm{mean}}}({\bf{A}},{\bf{B}})\le {{\mathrm{MPCS}}}_{{\mathrm{max}}}({\bf{A}},{\bf{B}})\le 1.$$

Applying the above-mentioned procedure to the toy example from Fig. 2 yields the MPCS values in Table 3. An example implementation of MPCS in Python is provided in the Supplementary Information (see Supplementary Fig. 1)

**Table 3:** MPCS values for the toy example from Figure 2

The cell highlighted in red indicates a violation of the *no-overlap* requirement, as it reveals that at least one CSSC is valid for both X and Y—that is, the two disorders share an identical criteria-satisfying symptom combination. This result aligns with the overlapping node colors (yellow and blue) in the Hasse diagram shown in Figure 2d.

### Sammon mapping

Sammon mapping is a nonlinear dimensionality reduction technique that projects high-dimensional data into a lower-dimensional space while preserving pairwise distances as faithfully as possible^32^. Unlike principal component analysis (PCA), which identifies linear components that maximize variance in the data, Sammon mapping prioritizes the preservation of pairwise distances, making it particularly suited for visualizing cluster structure in binary vector data such as CSSCs; in our setting, it outperformed both linear and nonlinear multidimensional scaling (MDS) in producing informative separations. The method minimizes Sammon’s stress function *E*, defined as:

9 $$E=\frac{1}{{\sum }_{i=2}^{n}{\sum }_{j=1}^{i-1}{d}_{ij}^{({\rm{o}}rig)}}\mathop{\sum }\limits_{i=2}^{n}\mathop{\sum }\limits_{j=1}^{i-1}{\left(1-\frac{{d}_{ij}^{({\rm{i}}mg)}}{{d}_{ij}^{({\rm{o}}rig)}}\right)}^{2}{d}_{ij}^{({\rm{o}}rig)}$$

where ${d}_{ij}^{({\rm{o}}rig)}$ and ${d}_{ij}^{({\rm{i}}mg)}$ denote the distances between objects *i* and *j* in the original and image space, respectively. Larger original distances are weighted more heavily, emphasizing the preservation of broader structural relationships.

The optimization is typically initialized using PCA coordinates and refined via gradient descent. The normalized stress *E* quantifies how well interpoint distances are preserved: lower values indicate better fidelity, with *E* = 0 representing perfect preservation.

### Generation method

In a first step, expert annotators manually translated the logic expressed in narrative DSM criteria into symptom combination generators ("generators”), which are predefined functions operating on structured symptom lists and thereby provide a formal representation of the criteria’s combinatorial logic (as illustrated in Fig. 1). To align with the binary structure of the model, duration thresholds and comparable contextual qualifiers were encoded as separate symptoms whenever they were prominent in the narrative criteria or explicitly described as such. In a second step, we applied these generators to systematically enumerate all criteria-satisfying symptom combinations (CSSCs) implied by the diagnostic criteria. A detailed and mathematically oriented description of the generator system—including the full formal specification of the generator types, their semantics, and implementation details—is provided in a companion methods preprint^33^; in the present manuscript, we focus on the resulting CSSCs and their analysis.

### Symptom harmonization

To support the formal analysis in Section *Newly emerging clinical conditions*, we harmonized the symptom terminology of Long COVID and comparator disorders to ensure representational consistency. Due to the string-based nature of our framework and the absence of standardized vocabulary terms at the symptom level, narrative descriptions from the 2024 NASEM report^22^ were systematically aligned with DSM-5-compatible phrasing. Symptom alignments are documented in the Supplementary Information (Section 4).

## Supplementary information

Supplementary Information

## Data Availability

The source data supporting the findings of this study, including the data used inTable 1 and Figure 3 and the associated calculated values, are available in a public GitHub repository at: https://github.com/raoul-k/AIDA-Path/tree/main/data.

## Code availability

The code used to generate the results in this paper is available in a public GitHub repository at: https://github.com/raoul-k/AIDA-Path. The code for generating CSSCs is publicly available at https://github.com/raoul-k/AIDA-Path/tree/main/Python/Generators, and the specific generators used in this document can be found at https://github.com/raoul-k/AIDA-Path/tree/main/data/generators. A Jupyter Notebook for testing the generators will also be made available in the same repository.
