# Supplementary material for: Machine-actionable criteria chart the symptom space of mental disorders
Source: NPJ Digit Med. 2026 Feb 23;9:271. doi: 10.1038/s41746-026-02451-6 (PMC13040010; doi:10.1038/s41746-026-02451-6)
Supplement: Supplementary file 1 — Supplementary Information [file 41746_2026_2451_MOESM1_ESM.pdf]

Supplementary Information for *Machine-actionable criteria chart the symptom space of mental disorders*

Strasser-Kirchweger, B
Kutil, RH
Zimmermann, G
Borgelt, C
Trutschnig, W
Hutzler F

Contents

1 Supplementary Note 1: Examples for core notions 2

2 Supplementary Note 2: Implementation of the MPCS algorithm 3

3 Supplementary Note 3: Computational considerations 4

4 Supplementary Note 4: Symptom harmonization examples 5

5 Supplementary Note 5: Example for the shared symptom space 6

6 Supplementary Note 6: From Narrative to Binary 7

1 Supplementary Note 1: Examples for core notions

A criteria-satisfying symptom combination is called *irredundant* (for a certain disorder) if and only if no proper subset of the symptom combination is criteria-satisfying. Otherwise it is called *redundant* (for that disorder). In case of the disorder W in Fig. 2 the CSSC consisting of symptoms a,b,c is redundant since the presence of a,b is also a valid CSSC (and the latter is irredundant and so is the CSSC consisting of a,c).
We call a symptom *relevant* for diagnosing a certain disorder if and only if there exists an irredundant criteria-satisfying symptom combination containing it. Otherwise the symptom is called *irrelevant* (for diagnosing that disorder). In case of the disorder W in Fig. 2, each of the symptoms a,b,c is relevant, whereas symptom d is irrelevant.
The term relevant here denotes logical rather than clinical relevance. A symptom is classified as relevant for a given disorder if it participates in at least one minimal symptom combination that is sufficient to satisfy that disorder's formal diagnostic criteria. In other words, each relevant symptom appears in a smallest possible set of symptoms that justifies the diagnosis in our formalisation; if that symptom were removed from that configuration, the remaining symptoms would no longer satisfy the criteria. We do not claim that this definition captures clinical importance or severity. It only captures whether a symptom can be logically necessary for the diagnosis in at least one minimal configuration.

A CSSC is called *weakly irredundant* (for a certain disorder) if and only if it contains only relevant symptoms (or, equivalently, no irrelevant symptoms). Otherwise it is called *strongly redundant* (for that disorder). In case of the disorder W in Fig. 2 the CSSC consisting of symptoms a,b,c is weakly irredundant (as are a,b and a,c). The CSSC containing all of symptoms a,b,c,d, however, is strongly redundant.

The diagnostic criteria of two disorders (or, shortly, two disorders) are called *separable* if, and only if there does not exist any symptom combination that is weakly irredundant (and hence criteria-satisfying) for both disorders. Otherwise they are called *overlapping.* Clearly, overlapping diagnostic criteria do not allow one to separate the corresponding disorders, even if one considers only relevant symptoms (and hence excludes comorbidities). The disorders X and Y, e.g., considered in Fig. 2 are overlapping, because the CSSCs bc and bcd are weakly irredundant for both X and Y.
The diagnostic criteria of a disorder X are said to *subsume* the diagnostic criteria of a disorder Y if there exists a symptom combination that is weakly irredundant (and hence criteria-satisfying) for disorder X and also criteria-satisfying (but not necessarily weakly irredundant) for disorder Y. For example, in Fig. 2 disorder W subsumes disorder Z, because the symptom set a,b (as well as the symptom sets a,c and a,b,c) is weakly irredundant (and hence criteria-satisfying) for disorder W, but also criteria-satisfying for disorder Z due to the presence of symptom a (though strongly redundant for Z, because it also contains b, which is irrelevant for Z).
Note that subsumption is *not* a symmetric concept: X subsuming Y does (in general) *not* imply Y subsuming X. Moreover, subsumption is weaker than overlap: If the diagnostic criteria of two disorders X and Y overlap, then X subsumes Y and Y subsumes X, while the reverse need not be true. Hence subsumption is a weaker—and directed—form of overlap of the diagnostic criteria of two disorders.

2 Supplementary Note 2: Implementation of the MPCS algorithm

Our implementation of MPCS uses a reversed row-column orientation of the matrix introduced in the main section. Adjusting the orientation of rows and columns reflects a more efficient algorithmic implementation, as many matrix operations benefit from having a larger number of rows than columns. Hence, the remaining paragraphs in this subsection use rows for the CSSCs and columns for the ordered symptoms.

We take advantage of Python's ability to efficiently handle matrix operations by computing all pairwise cosine similarities simultaneously. This is achieved through matrix-matrix multiplication combined with the outer product of the row-wise norms of both matrices. The outer product $A \otimes B$ of two vectors $A = (A_1, \ldots, A_n)$ and $B = (B_1, \ldots, B_m)$ is defined by

$$A \otimes B = AB^T = \begin{bmatrix} A_1 \\ A_2 \\ \vdots \\ A_n \end{bmatrix} [B_1, B_2, \ldots, B_m] = \begin{bmatrix} A_1B_1 & A_1B_2 & \cdots & A_1B_m \\ A_2B_1 & A_2B_2 & \cdots & A_2B_m \\ \vdots & \vdots & \ddots & \vdots \\ A_nB_1 & A_nB_2 & \cdots & A_nB_m \end{bmatrix} \tag{1}$$

where B^T denotes the transpose of the vector B.

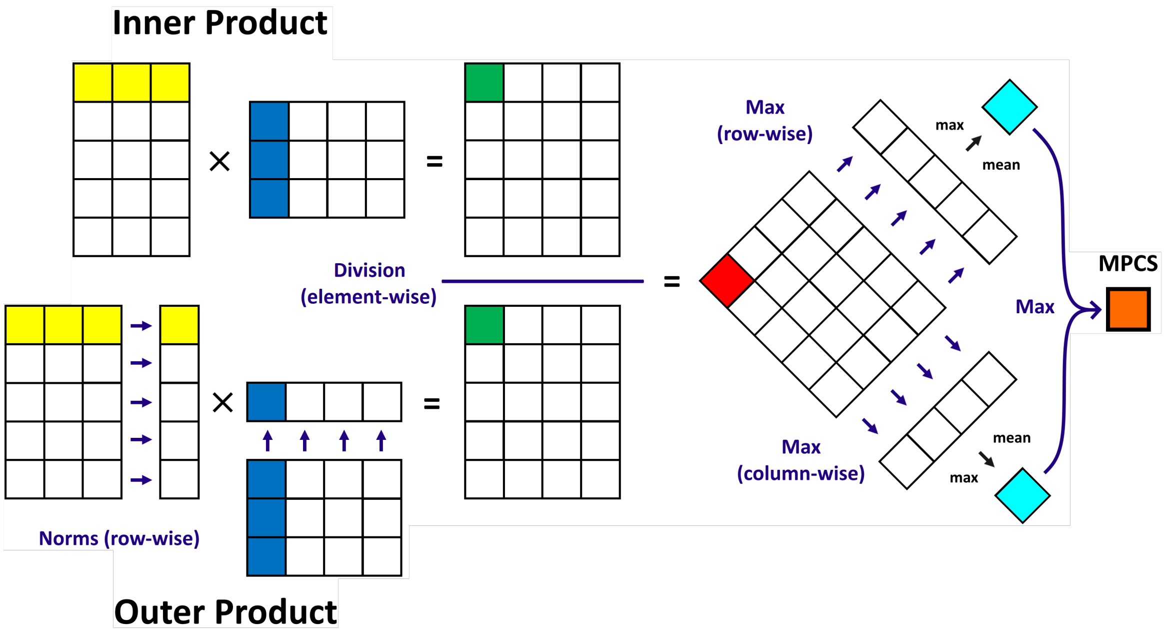

Supplementary Figure 1: Visualization of the MPCS algorithm implemented in Python.

The result is a similarity matrix with dimensions equal to the number of rows in matrix **B** by the number of rows in matrix **A**, enabling the element-wise (Hadamard) division between

matrices to obtain the cosine similarity values. In other words, we obtain a matrix in which every entry is the cosine similarity between a row vector from matrix **A** and one from matrix **B**. By taking the maximum of each column, we obtain the highest similarity for each row in **A**. Analogously, considering the maximum of each row yields the highest similarity for each row in **B**. Applying either the maximum or the mean aggregate to both of these lists, followed by calculating the maximum of the two values we finally obtain MPCS_{max} and $\text{MPCS}_{\text{mean}}$ (see Supplementary Figure 1).

3 Supplementary Note 3: Computational considerations

To document the practical feasibility of the CSSC generation procedure, we report runtime behaviour and hardware characteristics of our implementation. All simulations were conducted in Python on a desktop system running Windows 11 Enterprise, equipped with an Intel Core i5-8500 CPU (3.00 GHz) and 8 GB RAM. As representative examples, we recorded runtime and output size for Persistent Depressive Disorder (PDD) and for Criterion A of Major Depressive Disorder (MDD). In our implementation, the generation procedure for PDD produced a total of 63,567 CSSCs in 0.01 seconds, whereas for MDD Criterion A it produced 7,283,511 CSSCs in 164.91 seconds.

Assuming an approximately linear relationship between runtime and the number of generated CSSCs, this Criterion-A simulation suggests that a full implementation of all MDD criteria would require $7,283,511 \times 63$ (from Criterion B) $\times 3$ (from Criterion E) $= 1,376,583,579$ CSSCs and 9 hours of runtime on this hardware (2.75 minutes $\times$ 63 $\times$ 3 $\approx$ 9 hours). This estimate should be interpreted as a heuristic indication of scale rather than a precise bound, as runtime also depends on the specific generators used and the complexity of filtering valid CSSCs. To illustrate these generator-specific effects, Supplementary Table 1 compares three generator configurations for a disorder with 24 total symptoms (5 necessary symptoms, evenly distributed across several criterion items):

**Generator**	**CSSCs Generated**	**Runtime**
Generator 1	16,764,265	6 min 30 sec
Generator 2	16,588,509	9 min 43 sec
Generator 4	16,370,018	7 min 39 sec

Supplementary Table 1: Comparison of different generators in terms of total CSSCs generated and runtime.

These results show that, for the disorders considered in the present work, generating and filtering CSSCs is tractable on standard hardware, even for cases with tens of millions of combinations. Further optimisations (e.g., more efficient storage formats or generator refinements that reduce the number of intermediate profiles) are the subject of ongoing work and fall outside the scope of this paper.

4 Supplementary Note 4: Symptom harmonization examples

To align the Long COVID symptom definitions from the 2024 NASEM report with DSM-5 terminology, we performed a controlled mapping process. To illustrate, consider the Level 1 description from Ely et al. (2024, p. 1747): “Single or multiple symptoms, such as shortness of breath, cough, persistent fatigue, postexertional malaise, difficulty concentrating, memory changes, recurring headache, lightheadedness, fast heart rate, sleep disturbance, problems with taste or smell, bloating, constipation, and diarrhea.”

In our framework, this narrative description is translated into a formal requirement that at least one of the listed symptoms must be present, with each term harmonized to the DSM-5 symptom vocabulary where appropriate. The mapping involved the following systematic terminology adjustments to ensure consistency with DSM-5: “difficulty concentrating” was reformulated as “diminished ability to concentrate” (with “poor concentration” as a synonym); “lightheadedness” was supplemented with “feeling light-headed”; “hypersomnia” and “insomnia” were grouped under “sleep disturbance”; “fast heart rate” became “accelerated heart rate”; and gastrointestinal symptoms (“bloating,” “constipation,” “diarrhea”) were aggregated under the DSM-5 term “abdominal distress.” Only symptoms explicitly mentioned in the NASEM report were included in this mapping.

In parallel, we applied a set of principled exclusions when encoding DSM-5 criteria for the disorders used in our analyses. To avoid diluting disorder-specific signal, we did not encode non-differentiating DSM-5 criteria such as “The episode is not attributable to the physiological effects of a substance or to another medical condition” (which applies to all disorders considered) and “The symptoms cause clinically significant distress or impairment in social, occupational, or other important areas of functioning” (excluded from Major Depressive Disorder, Persistent Depressive Disorder, and Generalized Anxiety Disorder in our formalisation). A descriptive clause in PDD—“Criteria for a major depressive disorder may be continuously present for two years”—was also excluded, as it is not a formal requirement.

5 Supplementary Note 5: Example for the shared symptom space

To make cross-disorder comparisons possible, CSSCs from different disorders must be embedded in a shared symptom space. The following toy example illustrates this embedding step for two hypothetical disorders with partially overlapping symptom sets, showing how their disorder-specific CSSCs are represented in a shared space before similarity and delineation are evaluated.

Assume Disorder 1 with the relevant symptoms {S1, S2, S3, S4, S5} and the following CSSCs:

**CSSC**	**S1**	**S2**	**S3**	**S4**	**S5**
{S1, S2, S3, S4}	1	1	1	1	0
{S1, S2, S4, S5}	1	1	0	1	1
{S1, S2, S3, S4, S5}	1	1	1	1	1

Assume Disorder 2 with the relevant symptoms {S3, S5, S6, S7, S8} and the following CSSCs:

**CSSC**	**S3**	**S5**	**S6**	**S7**	**S8**
{S3, S5, S7, S8}	1	1	0	1	1
{S5, S6, S7, S8}	0	1	1	1	1
{S3, S5, S6, S7, S8}	1	1	1	1	1

To embed both disorders in a shared symptom space, we take the union of their relevant symptoms, {S1, S2, S3, S4, S5, S6, S7, S8}. Each disorder's CSSCs are then extended to this space by adding any previously absent symptoms as additional columns and assigning a value of zero to the corresponding cells (highlighted in red below).

Disorder	**CSSC**	**S1**	**S2**	**S3**	**S4**	**S5**	**S6**	**S7**	**S8**
1	{S1, S2, S3, S4}	1	1	1	1	0	0	0	0
	{S1, S2, S4, S5}	1	1	0	1	1	0	0	0
	{S1, S2, S3, S4, S5}	1	1	1	1	1	0	0	0
2	{S3, S5, S7, S8}	0	0	1	0	1	0	1	1
	{S5, S6, S7, S8}	0	0	0	0	1	1	1	1
	{S3, S5, S6, S7, S8}	0	0	1	0	1	1	1	1

6 Supplementary Note 6: From Narrative to Binary

Here we provide a toy example that illustrates the same two-step process described in the main Methods section: first, narrative diagnostic criteria are encoded as explicit symptom sets (represented in practice via generators), and second, these encodings are used to generate all criteria-satisfying symptom combinations (CSSCs) and embed them as binary vectors. The starting point is a hypothetical disorder whose diagnostic criteria are expressed in narrative form:

Disorder 1 - Diagnostic Criteria

A. At least one of the following symptoms:
   1. Symptom 1
   2. Symptom 2

B. One (or more) of the following symptoms:
   1. Symptom 3
   2. Symptom 4 or Symptom 5

C. The disorder is not better explained by Disorder 2 or Disorder 3.

Criterion A requires "at least Symptom 1 or Symptom 2"; Criterion B requires "presence of one or more of the following: Symptom 3; Symptom 4 or Symptom 5"; and Criterion C states that the presentation is "not better explained by Disorder 2 (Symptom 6) or Disorder 3 (Symptom 7)." For this example, we consider a finite symptom inventory of seven binary features, S_1–S_7, where S_1–S_5 denote the primary symptoms and S_6 and S_7 encode the two "not better explained by ..." conditions.

In the first step, we identify, for each criterion, the minimal symptom sets that satisfy it. For Criterion A ("at least Symptom 1 or Symptom 2"), one of the two symptoms is present, with co-occurrence allowed. This yields three admissible symptom sets:

Symptom sets for A: {S1}, {S2}, {S1, S2}

For Criterion B ("one or more of: Symptom 3; Symptom 4 or Symptom 5"), we first treat "Symptom 4 or Symptom 5" analogously as "at least one of $S4$ and $S5$", and then enumerate all minimal sets that contain at least one of $\{S3, S4, S5\}$. This results in:

Symptom sets for B: {S3}, {S4}, {S5}, {S4, S5},
{S3, S4}, {S3, S5}, {S3, S4, S5}

Criterion C is treated as a single condition that is satisfied only when both exclusion clauses hold, yielding:

Symptom sets for C: {S6, S7}

In the full framework, these collections of symptom sets are represented compactly using the generator types described in Kutil et al. (2025, arXiv:2511.18645); here we list them explicitly for illustration.

In the second step, we obtain the full set of criteria-satisfying symptom combinations (CSSCs) by systematically combining one admissible set from A, one from B, and one from C, and for each such triple forming the union of its elements. Each union yields one CSSC. For example, combining $\{S1\}$ from A, $\{S3\}$ from B, and $\{S6, S7\}$ from C yields the CSSC $\{S1, S3, S6, S7\}$. Each row in the lower table corresponds to one such CSSC.

Finally, we embed these CSSCs into a binary matrix by ordering the symptoms as $(S1, \ldots, S7)$ and coding symptom presence as 1 and absence as 0, which produces the binary representation shown in the right-hand columns. Together with the example in Section 5 (embedding disorders into a shared symptom space), this worked example makes explicit how narrative criteria are first encoded as formal symptom sets (via generators) and then systematically expanded into a complete, binary CSSC representation:

**CSSC (A×B×C)**	**S1**	**S2**	**S3**	**S4**	**S5**	**S6**	**S7**
S1, S3, S6, S7	1	0	1	0	0	1	1
S1, S4, S6, S7	1	0	0	1	0	1	1
S1, S5, S6, S7	1	0	0	0	1	1	1
S1, S3, S4, S6, S7	1	0	1	1	0	1	1
S1, S3, S5, S6, S7	1	0	1	0	1	1	1
S1, S4, S5, S6, S7	1	0	0	1	1	1	1
S1, S3, S4, S5, S6, S7	1	0	1	1	1	1	1
S2, S3, S6, S7	0	1	1	0	0	1	1
S2, S4, S6, S7	0	1	0	1	0	1	1
S2, S5, S6, S7	0	1	0	0	1	1	1
S2, S3, S4, S6, S7	0	1	1	1	0	1	1
S2, S3, S5, S6, S7	0	1	1	0	1	1	1
S2, S4, S5, S6, S7	0	1	0	1	1	1	1
S2, S3, S4, S5, S6, S7	0	1	1	1	1	1	1
S1, S2, S3, S6, S7	1	1	1	0	0	1	1
S1, S2, S4, S6, S7	1	1	0	1	0	1	1
S1, S2, S5, S6, S7	1	1	0	0	1	1	1
S1, S2, S3, S4, S6, S7	1	1	1	1	0	1	1
S1, S2, S3, S5, S6, S7	1	1	1	0	1	1	1
S1, S2, S4, S5, S6, S7	1	1	0	1	1	1	1
S1, S2, S3, S4, S5, S6, S7	1	1	1	1	1	1	1
